# Influence of Nanosilica and PVA Fibers on the Mechanical and Deformation Behavior of Engineered Cementitious Composites

**DOI:** 10.3390/polym17152067

**Published:** 2025-07-29

**Authors:** Mohammed A. Albadrani

**Affiliations:** Department of Mechanical Engineering, College of Engineering, Qassim University, Buraydah 51452, Saudi Arabia; moa.albadrani@qu.edu.sa

**Keywords:** deformation characteristics, Engineering Cement Composite (ECC), nanosilica, polyvinyl alcohol (PVA) fibers, sustainability, mechanical properties, Abaqus simulation

## Abstract

This paper evaluates the synergistic effect of polyvinyl alcohol (PVA) fibers and nanosilica (nS) on the mechanical behavior and deformation properties of engineered cementitious composites (ECCs). ECCs have gained a reputation for high ductility, crack control, and strain-hardening behavior. Nevertheless, the next step is to improve their performance even more through nano-modification and fine-tuning of fiber dosage—one of the major research directions. In the experiment, six types of ECC mixtures were made by maintaining constant PVA fiber content (0.5, 1.0, 1.5, and 2.0%), while the nanosilica contents were varied (0, 1, 2, 3, and 5%). Stress–strain tests carried out in the form of compression, together with unrestrained shrinkage measurement, were conducted to test strength, strain capacity, and resistance to deformation, which was highest at 80 MPa, recorded in the concrete with 2% nS and 0.5% PVA. On the other hand, the mixture of 1.5% PVA and 3% nS had the highest strain result of 2750 µm/m, which indicates higher ductility. This is seen to be influenced by refined microstructures, improved fiber dispersion, and better fiber–matrix interfacial bonding through nS. In addition to these mechanical modifications, the use of nanosilica, obtained from industrial byproducts, provided the possibility to partially replace Portland cement, resulting in a decrease in the amount of CO_2_ emissions. In addition, the enhanced crack resistance implies higher durability and reduced long-term maintenance. Such results demonstrate that optimized ECC compositions, including nS and PVA, offer high performance in terms of strength and flexibility as well as contribute to the sustainability goals—features that will define future eco-efficient infrastructure.

## 1. Introduction

Numerous obstacles have arisen due to the wide range of materials currently available and the desire to improve performance or develop new materials for specific purposes. Numerous building materials have developed quickly as a result of new materials’ enhanced efficiency and sophistication. To optimize their use and guarantee their anticipated performance over time, material durability needs to be routinely examined [1]. Given the urgent need to reduce the carbon footprint of the built environment in response to climate change, engineered cementitious composites (ECCs) have become a topic of significant interest as environmentally sustainable alternatives to conventional concrete [2]. ECCs reduce the environmental load by decreasing maintenance requirements resulting from the corrosion of steel and cracking of concrete in reinforced structures [3]. Nevertheless, despite these advantages, ECCs may have greater embodied energy and carbon emissions than ordinary concrete, as they contain high amounts of cement and fibers. This has prompted researchers to identify alternative materials, including modified sands, fibers, and additional binders, to maintain the same mechanical performance and durability of ECC while enhancing sustainability [4].

ECCs are composite materials composed of cement, distinguished by their high ductility, strain-hardening ability, and adjustable fiber reinforcement, when compared to typical concrete [5]. Their tensile strain capacity can be hundreds of times larger than that of normal concrete, mainly due to controlled microcracking that occurs in tension. Mineral additives, such as fly ash (FA) and granulated blast furnace slag (GBFS), have been successfully used to enhance workability, durability, and cost efficiency [6]. However, variations in ECC performance between small-scale laboratory production and large-scale field applications have been attributed to mixer type, mixing procedure, and local material characteristics [7].

Despite the excellent tensile properties of ECCs, prior studies demonstrated that a decrease in fiber content or the substitution of fibers with other materials can compromise the mechanical behavior, including tensile strength and ductility [5]. To overcome this problem, recent studies examined how nanotechnology can enhance the performance of ECC [8]. Nanomaterials, such as nanosilica (nS), have also been proven to be promising in optimizing cementitious composites, aiming to improve microstructure and performance [9]. Nanoparticles promise a prospect of a permanent drop in costs, better ecological operation, and extended structural service life [10]. Specifically, Haruna (2023) [11] showed that the molecular nano-modification improves the mechanical and microstructural properties of ECC when used in infrastructure (buildings and bridges). Adding Al_2_O_3_ and TiO_2_ nanoparticles has also resulted in notable increases in compressive strength, with an increase of more than 26% and 34%, respectively, even at high temperatures [12]. Furthermore, Öztürk et al. (2020) [13] established that the nS/multiwalled carbon nanotube (MWCNT) additive improves self-healing and sensing performance of ECC due to the denser calcium–silicate–hydrate (C–S–H) and CaCO_3_ formations in the healed cracks.

Additional research indicated that nS enhances matrix density, rapid cement hydration, impermeability, and durability [14]. Graphene oxide (GO) has also shown promise in ECC applications, with one article reporting a 30% increase in compressive strength and improved sulfate and acid resistance upon the addition of 0.05% GO to 1% PVA fibers [15]. The recovery rate of tensile strength reached 131.46% in an ECC formulation containing 0.75% nS (Ahmed, 2023) [16], demonstrating the importance of nanoparticles in enhancing self-healing and extending longevity of the material.

Simultaneously, the contribution of PVA fibers in ECC has been determined under various conditions. For example, Tanyldiz (2024) [17] investigated the self-healing ability under high temperature with carbon nanotubes and found the maximum healing rates at 200 °C. Nevertheless, subsequent additions of nanoparticles can diminish workability due to the entrapping of water and large surface area effects, as noted by Chadha (2024) [18]. Such a confirmation concerning the improvement of matrix density and hydration, with a decline in flexibility, was made by Jakhar (2024) [19] in the context of the nanotechnological advancement.

Despite these developments, a distinct gap remains in understanding the synergistic impact of nS and PVA fibers on the deformation and mechanical behaviors of ECC. Most research has been conducted separately on nanoparticles or fibers, but there is limited information on the synergistic effect of compressive strength and strain behavior at different mix proportions.

Additionally, there is limited discussion on the economic viability of reducing fiber volume through the incorporation of nS. The present study addresses this gap by investigating a wide range of ECC formulations with varying nS and PVA contents, using experimental methods to evaluate their mechanical response, deformation behavior, and potential for more sustainable, cost-efficient ECC design.

## 2. Materials and Methods

**Figure 1:**
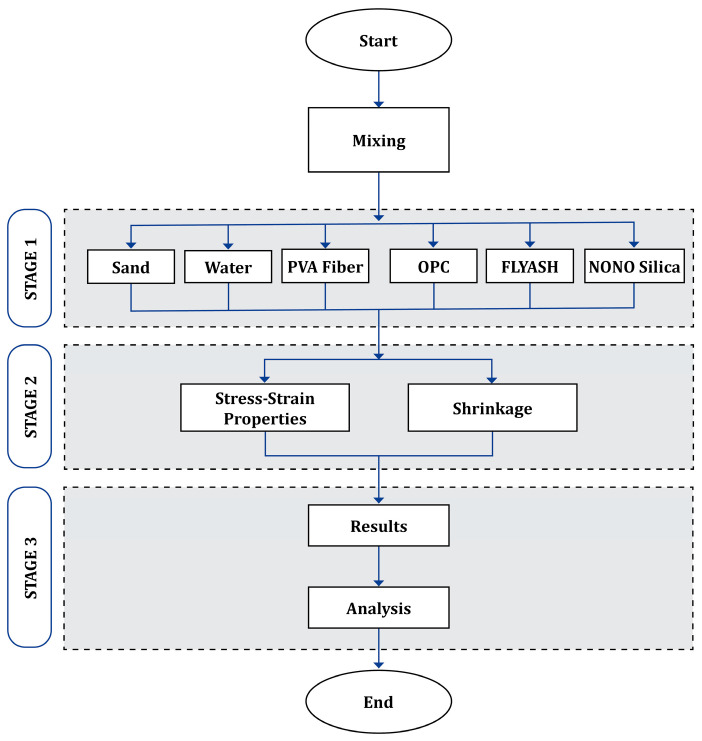
The phases of this study.

In Figure 1, the research technique is illustrated. It is separated into the following three stages:

- Material preparation and mixture testing for the sample.
- Testing the samples.
- Examining the test results obtained.

### 2.1. Preparation for the Samples

This section outlines the stages of sample preparation. It presents the materials used in producing ECC, along with their descriptions and properties, and details the ECCs with varying nS mixing proportions.

This study aimed to assess the effect of nS particles and polyvinyl alcohol (PVA) fibers on compressive and mechanical properties of engineered cementitious composites (ECCs) (Table 1). Experimental mix designs were formulated using the known ranges reported in the literature [7,8,9,10,19,20,21,22] and in accordance with ASTM C192/C192M-19 [23]. Namely, the amount of PVA fibers added ranged between 0.18 kg and 0.54 kg (or roughly 0.5% to 2% of the dry material weight), and that of nS content ranged from 0% to 5%. Both PVA fibers and nS have been proven to provide significant performance enhancements in prior studies. The present research was restricted to nS contents ranging from 0% to 5%, which is widely reported in the literature as the most effective concentration in enhancing compressive strength and decreasing shrinkage without any negative impact on the mix operation or leading to excessive requirements of chemical additives. Other researchers, including Qing et al. (2007), verified that adding more nS than this limit may actually cause a rise in viscosity and agglomerate formation, which adversely affects the fine particle distribution and ultimately impairs structural performance instead of enhancing it [2].

This study involved a controlled and repeatable experiment to measure the mechanical and sustainability capacity of ECCs reinforced with polyvinyl alcohol (PVA) fibers and different proportions of nS. Ordinary Portland Cement (OPC) of ASTM Type I, Grade 43, was considered the primary cementitious binder, supplemented with fine-grade fly ash to enhance long-term durability and rheology of the mix. The fine aggregate used was natural sand that met the requirements of BS 882:1992. PVA fibers, with a tensile strength of 1620 MPa and an average length of 12 mm, were added to improve the ductility and crack resistance. In contrast, amorphous nS particles, with an average diameter of 19 nm and a specific gravity of 2.12 g/cm^3^, were included to densify the matrix and increase the strength properties. To achieve the desired workability, superplasticizer (SP) was incorporated into different mixes in varying proportions, with Conplast SP430A1 being added in the range of 0.27–0.77 kg. The mix design consisted of a constant weight of OPC (17.6 kg), sand (13.9 kg), fly ash (20.8 kg), and water (5.5 kg) in quantity, whereas the volume of PVA and nS was altered in accordance with the requirements listed in Table 2.

Mixing was initiated by dry blending OPC, fly ash, nS, and sand over 2 min, followed by the addition of water, which was slowly added over 1 min. Then, SP was added, and, lastly, PVA fibers were dispersed into the mixture. The entire mixing process took place between 5 and 10 min to create uniformity. Mixing and curing were performed under fixed environmental conditions: the temperature ranged between 1824 °C, and the relative humidity was greater than 95%.

Table 3 and Table 4 show the main properties of the material used in the experimental ECC mixtures. The mechanical and physical properties of PVA fibers are summarized in Table 3 and Table 4, which provide the composition and specifications of the nanosilica additive. The use of Polyvinyl Alcohol (PVA) fibers was considered, more particularly because of their high tensile strength, ductility, and excellent bonding properties in cementitious composites, particularly in ECCs [12]. Unlike normal concrete they are common in high-performance composites; however, PVA fibers are not standard in normal concrete. They have been reported to resist alkaline conditions [22] principally when surface-treated. Because of the scale constraints, there was no direct alkali degradation testing performed, but there was use of alkali-resistant, commercial-grade PVA fibers. This guarantees durability and mechanical competence in long-term cementitious conditions.

**Figure 2:**
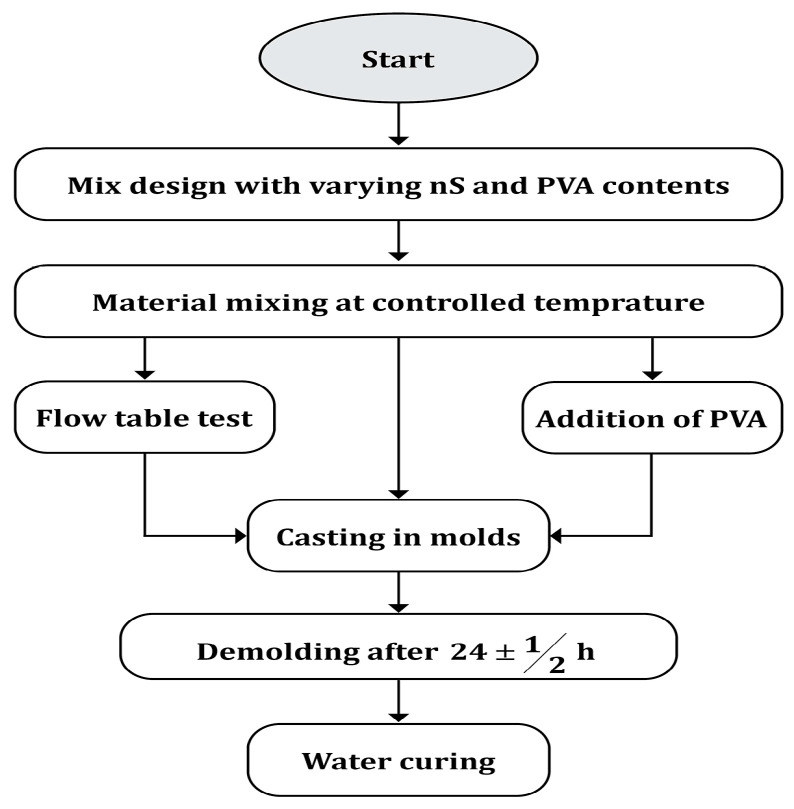
Flowchart of the sample preparation and mixing process used in this research.

The mechanical and deformation properties of the ECC mixtures were found to be strongly reliant on the constituent material composition and proportions. Thus, the subsections dedicated to materials, mix design, and experimental methods are structured. Additionally, the dosage of SP (Conplast SP430A1) was optimized based on the flowability needs determined by the flow diameter at the flow table test, in accordance with ASTM C1437. The desired flow diameter was set between 170 mm and 220 mm to ensure consistency of workability among all mixes. SP was added gradually up to this range. All the samples were poured into standard molds and allowed to cure for 28 days in a controlled water tank at a temperature of 23 ± 2 °C and a relative humidity exceeding 95%. These conditions have been selected to ensure uniform hydration and promote microstructural growth. The sample preparation and mixing process, which constitutes stage 1 of this research phase (refer to Figure 1), is illustrated in the flowchart depicted in Figure 2.

To ensure uniform distribution of the PVA fibers and prevent clumping within the mix, a common problem due to their physical properties, they were added manually and slowly during the final mixing phase. The fibers were gradually sprinkled onto the surface of the mix as it rotated in the mixer, allowing for even distribution without clumps or lumps, thus improving the overall mechanical performance of the concrete Table 2. The number of samples (*n*) used for each experimental mix in the Methodology Section is 4.

**Figure 3:**
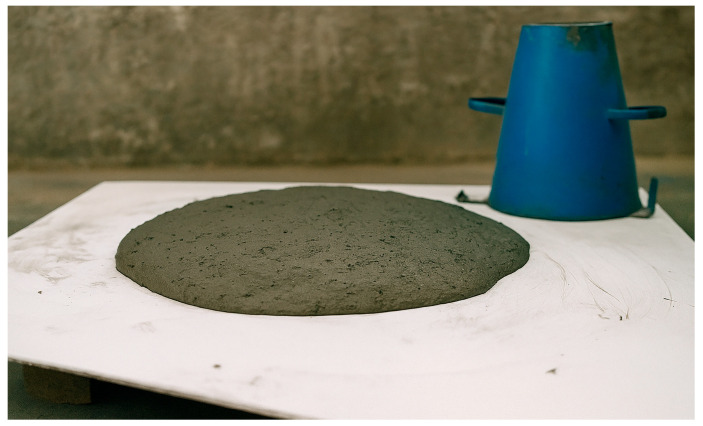
Concrete workability test and the used concrete molds.

**Figure 4:**
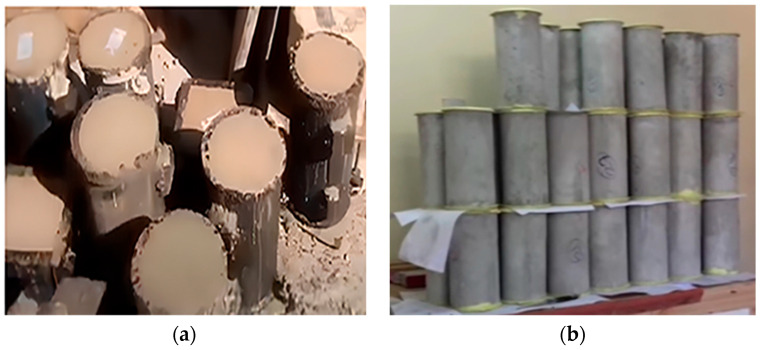
(**a**) Before testing molded samples, (**b**) samples are allowed to cure for 28 days.

For specimen preparation, all the dry materials were first blended in a concrete mixer for 1–2 circular revolutions. It was prepared in a separate bucket and then gradually poured into the dry mixture to prevent coagulation. Then, water and SP were added to the dry mixture to complete the mixture. The entire mixing process took approximately 5–10 min. Finally, a flow test was conducted to ensure uniformity in workability for all mixtures, and more SP was added to the mix, where necessary. The prepared mixture was then poured into iron molds with a cross-section corresponding to the required sample size, as specified in ASTM C192/C192M-19 [24]. Figure 3 displays the ECC sample preparations. After 24 h, the samples were removed from the molds and transferred into a curing tank, where they were allowed to cure and hydrate for 28–30 days (Figure 4).

### 2.2. Testing Procedures

The test protocols for ECC samples are presented in this section. It provides standard test methods for the strain-and-stress analysis of concrete under compression, as well as for the length change in hardened hydraulic-cement mortar and concrete. To test compressive strength, specimens were cast in cylindrical and cuboid shapes with a 50 mm diameter and 100 mm height. For shrinkage testing, beam specimens were cast with a 25 mm square cross-section and a 285 mm length, in compliance with ASTM C192/C192M. All specimens were demolded after 24 ± 0.5 h and subjected to curing in lime-saturated water for up to 30 days.

**Figure 5:**
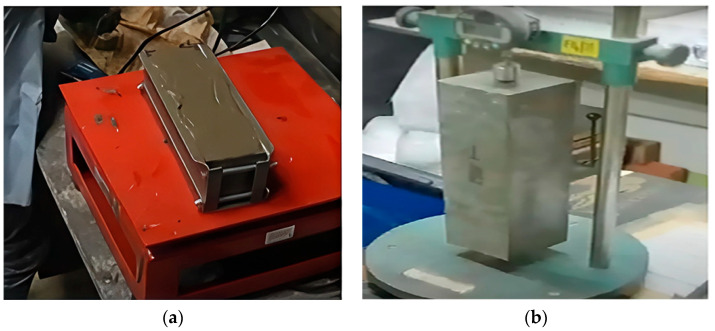
Shrinkage sample and the tester: (**a**) during mold, (**b**) during test.

To assess the shrinkage properties of ECC, the initial length of all samples was measured and then compared with their final length after curing for 28–30 days. They used the specimens by preparing a 25 mm square cross-section, approximately 285 mm in length, and mixed them at a constant temperature of 18–24 °C, as specified in ASTM C192/C192M. In cases where the molds had two halves, each half was machined halfway, padded, and then leveled with a trowel. After 24 ± ½ h, the samples were demolded and placed in lime-soft water for at least 15 min before measuring the quantity of hardened concrete using a comparator. These specimens were also left in the same water until the 28th day to take major measurements of the test, as depicted in Figure 5. All specimens were cylindrical and of uniform size. No variation in shape or dimensions was observed; thus, results are solely attributed to material changes, not geometry.

For stress–strain analysis, cylindrical ECC specimens with a 100 mm × 200 mm cross–sectional area was prepared using a fresh concrete mix, following ASTM C192/C192M. Samples were loaded using a loading frame, and while applying load, strain was measured using strain gauges. This test output was recorded via a data logger, and the stress was derived from the obtained load distribution. All this collected information was quite useful in determining stress–strain loops as well as in understanding the behavior of the material under compressive force.

### 2.3. Abaqus Procedures

**Figure 6:**
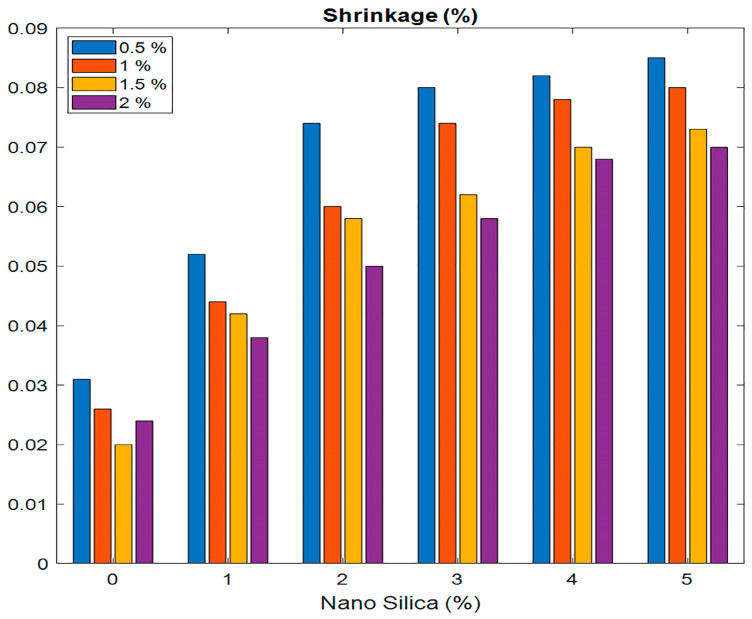
Relationship between nanosilica and shrinkage (%) for PVA fibers.

This study presents a finite element analysis (FEA) of a uniaxial compression test performed on a concrete cylinder using ABAQUS/CAE software, version 2020. The primary objective was to replicate an experimental compression test and validate the numerical model by comparing simulation results with experimental data. A concrete cylinder with dimensions of 100 mm in diameter and 200 mm in height was modeled. The concrete damaged plasticity (CDP) model was employed to simulate the nonlinear behavior of concrete under compression. A mesh size of 10 mm was used, employing C3D8R elements (8-node linear brick elements with reduced integration). The 10 mm mesh size was chosen based on a preliminary sensitivity study, which tested the effect of reducing the mesh size on the accuracy of the results versus increasing computation time. C3D8R was selected because it has demonstrated promising results in CDP-based modeling of concrete, particularly when the goal is to capture the global behavior (e.g., load–displacement, average stress–strain response) rather than detailed crack propagation or failure patterns. For example, the CDP model was originally developed and validated by Lee and Fenves (1998) using C3D8R elements, which demonstrated the sufficiency of these elements in reproducing damage development in concrete under cyclic and monotonic loading [25]. Furthermore, the latest comparative research, including the article by García and Reyes (2019) and Ranjbar et al. (2021), demonstrates that C3D8R elements strike a decent balance between precision and computational cost, particularly when the mesh size is chosen carefully [26,27]. These studies found that although higher-order or incompatible mode elements (e.g., C3D8I, C3D20R) can provide a better local strain resolution, C3D8R can give good results in predicting compressive behavior, damage initiation, and post-peak softening so long as mesh sensitivity is checked and appropriate controls (e.g., hourglass stabilization) are employed. Based on this, in the present simulation, the study was grounded on the C3D8R element to define the scope and scale of the simulation (Figure 6). Based on Abaqus simulation, the parameters were specified as follows: elastic modulus (22.4 GPa), Poisson’s ratio (0.20), compressive strength (43.2 MPa), tensile strength (4.2 MPa), density (2250 kg/m^3^), dilation angle (35°), flow potential eccentricity (0.1), the biaxial-to-uniaxial compressive strength ratio (1.16), and a viscosity parameter (0.0001). Boundary conditions were applied by fixing the base of the cylinder and applying a displacement-controlled load at the top surface.

The CDP model was used to accurately describe how ECC behaves when it is compressed in a nonlinear way. To make sure that the softening regime converges, key parameters were calibrated using experimental data. These included a dilation angle of 40°, a flow potential eccentricity of 0.1, and a viscosity parameter of 0.001. Progressive failure and ductility were simulated in Abaqus by adjusting CDP damage evolution and stiffness degradation to reflect the synergy of fibers and nS.

## 3. Results and Discussion

This section presents the results and discussion of the study on the influence of nS and PVA fibers on the mechanical and deformation behavior of ECC, including mechanism analysis and microstructural interpretation, as well as the environmental and sustainability performance of modified ECC.

### 3.1. Influence of Nanosilica and PVA Fibers on the Mechanical and Deformation Behavior of ECC

When analyzing the shrinkage percentages in ECC mixtures containing different levels of nS and PVA fibers, it can be observed that as the nS percentage increases from 0% to 5%, the shrinkage percentages of the ECC mixtures decrease across all levels of the PVA fiber content. These percentages are obtained by calculating the proportion of each component relative to the total binder weight. The results demonstrated that the incorporation of nS leads to a decrease in shrinkage, a modification of the cement matrix microstructure, which allows for an improvement in its hydration condition and ultimately fills the microvoids. This results in a more compact composite with a reduced tendency to undergo dimensional variations. PVA fibers play a decisive role in decreasing shrinkage, which in turn strengthens the composite and, therefore, prevents the spread of microcracks. This is evidenced by the fact that concrete with PVA fibers of 1.5% to 2.0% reduces the shrinkage percentage compared to the control concrete with no fibers. Additionally, the overall results of the experiment suggest that incorporating both PVA fibers and nanosilica results in reduced shrinkage; however, any incremental addition of these materials yields only marginal reductions in shrinkage. These results show that incorporating 3–4% nS with 1.0–1.5% PVA fibers reduces shrinkage by a greater percentage while enhancing the material’s efficiency. This combination provides the best outcome for shrinkage and reduces cracking in structural applications. The overall decrease in shrinkage experienced when nanosilica and PVA fibers are incorporated into the concrete structure is conducive to the overall sustainability of concrete structures. A lower shrinkage also decreases the likelihood of early-age cracking and improves the service life of the structure, making it ideal for roads, bridges, and buildings that require less maintenance but high performance. The addition of nS to ECC mixtures exhibited a complicated effect on the shrinkage behavior. At intermediate dosages, nS helped reduce shrinkage, which could be attributed to the enhanced packing density, pozzolanic reactions, and the integrity of the matrix, as corroborated by studies [20,21,22,23,24,25,26,27,28,29]. However, when the nS content was higher (especially when the volume of PVA fibers was greater), shrinkage would somewhat increase (Figure 6). This implies that the positive contribution of nS in mitigating shrinkage can level off or even become reversible once the matrix becomes over-dense or when internal autogenous shrinkage is induced by enhanced surface area and hydration kinetics. This makes ECCs a prospective material that could be used in sustainable constructions and for structures that require high resilience (see Figure 6 and Table 5).

**Figure 7:**
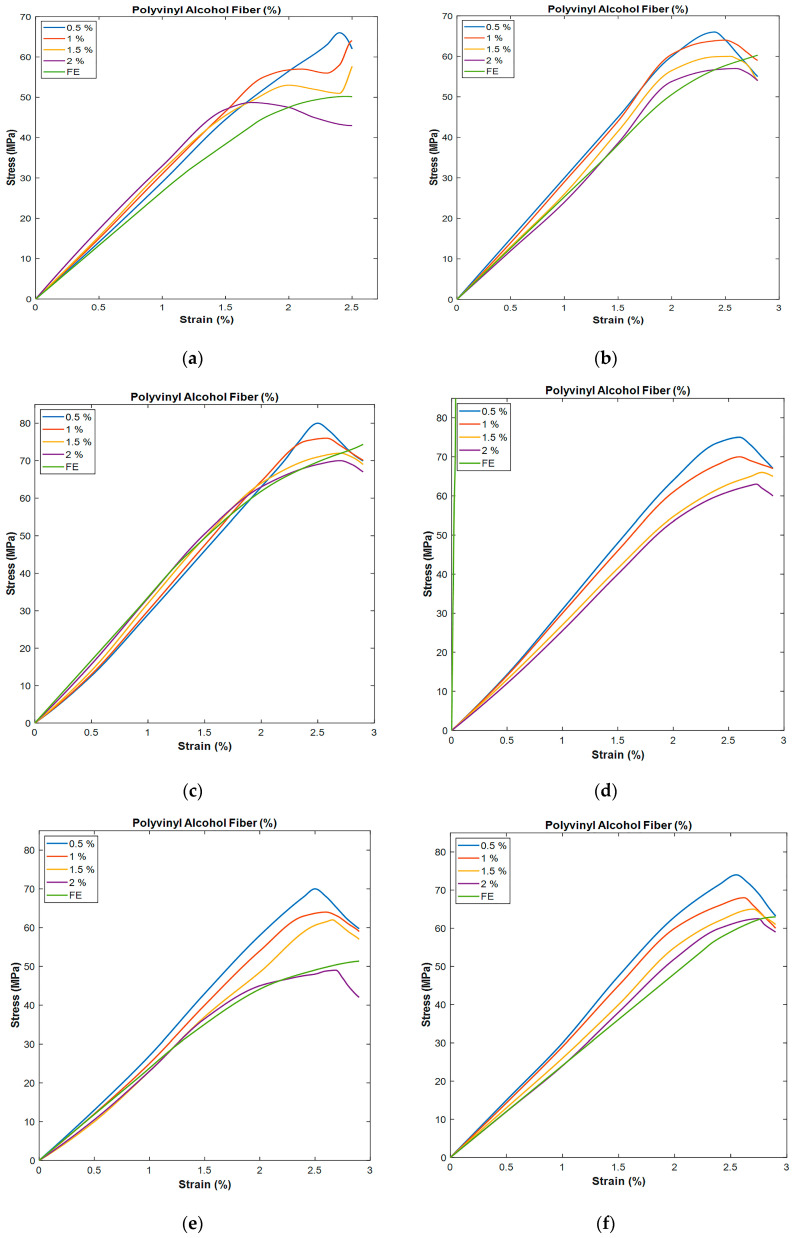
Relationship between stress and strain for PVA percentages: (**a**) 0%, (**b**) 1%, (**c**) 2%, (**d**) 3%, (**e**) 4%, and (**f**) 5% nS.

Figure 7 reveals that as the fiber content increases, the stress–strain curve also generally shifts upward, indicating higher stress capacities with higher fiber content. By focusing on the effect of nS, Figure 7a shows that the maximum stress, approximately 60 MPa, occurs after stretching the material to about 2% without the addition of nS. For each increasing PVA fiber percentage curve, the inflection point of the strain is approximately 3% at 65 MPa. Of the four PVA fiber curves presented, the 2% PVA fiber curve was the highest and widest, with a reinforcement ratio of fibers that was slightly effective due to the absence of nS additives. In Figure 7b, which represents the findings on the 1% nS, the maximum tensile stress elastic limit reaches 65 MPa, with strain abruption at 2.3%. This could possibly illustrate that such PVA fibers, when enhanced with nS ratios above 1%, yield the possible integration of fiber and matrix interfacial adhesion. Peaking at approximately 80 MPa at nearly 2.5% of the top range, the effect of increasing PVA in higher percentages results in a distinctive incremental trend. Notably, the 2% nS exhibited stronger adhesive properties between the fibers and the matrix, leading to more substantial interfacial cohesion effects, which enabled the material to withstand higher stress. The 3% nS exhibits the highest ultimate stress among all samples, indicating that this concentration offers optimal reinforcement under the specified conditions. Contrarily, Figure 7d displays that for the 4% nS, the ultimate stress is slightly lower than that in the 3% case, suggesting that increasing nS content to 4% does not necessarily enhance strength beyond the 3% level (Figure 7e). The ultimate stress in the 5% nS decreases further compared to the 3% and 4% levels. This suggests that the addition of 5% nS may lead to reduced efficiency in reinforcing the PVA fibers, possibly due to particle agglomeration. Hence, 3% nS provided the best results, as it was able to provide the necessary reinforcement, with stress values being the highest across all fiber contents. It was found that a combination of 3% nS and 2% fiber content provided the best ultimate stress and ultimate strain, possibly due to a synergistic effect. It is worth noting that incorporating more than 3% nS can lower the effectiveness of reinforcement due to expected agglomeration, thereby weakening the composite.

**Figure 8:**
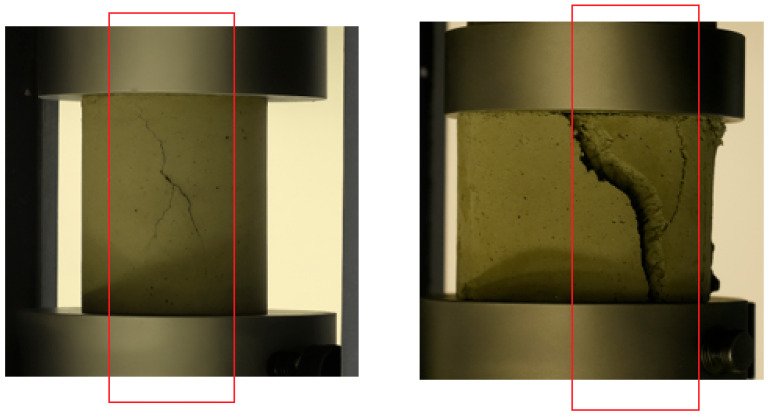
ECC sample under compression test.

**Figure 9:**
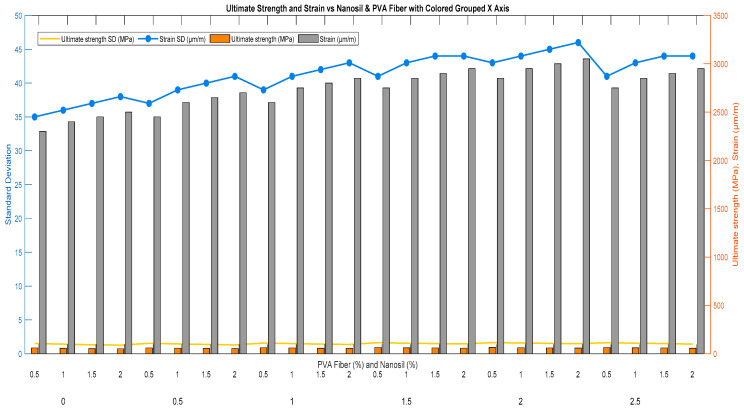
Effect of nanosilica and PVA fiber content on ultimate strength and strain of ECC mixtures.

Table 5 and Figure 8, Which show the ECC sample under a compression test, and Figure 9 demonstrate the correlation of ultimate strength (MPa) and strain (µm/m) of ECC mixes containing several percentages of nS and PVA fibers. These results prove that the addition of nS significantly increases the ultimate strength of the ECC. The highest strength is obtained at 2% nS, where the enhancement of bonding and the microstructure of the cement matrix is increased (up to 80 MPa with 0.5% PVA fiber).

However, at higher nS content (4% and 5%), the ultimate strength was observed to degrade. This is indicative of the fact that, after a certain level, the advantage of nS cannot be obtained, possibly due to an agglomeration effect or workability problems. The maximum load routinely rises with concentrations of nS and PVA fiber contents. This shows enhancement in the ductility of the ECC to be superior to that made without the incorporation of the said fiber.

A maximum of 2750 µm/m is reached from the mixture containing 3% nS with 1.5–2.0% PVA fiber and 5% nS with 2.0% PVA fiber. This suggests that integrating these materials enhances the strain hardening characteristics, which are crucial in controlling failure and facilitating greater energy absorption.

Therefore, the optimum proportion for providing strength while minimizing strain was found to be approximately 2–3% nanosilica with 0.5–1.5% PVA fibers. Together, they offer high strength and improved strain capability, which are desirable characteristics in structural members. The best results in ECC can be achieved by carefully balancing quicker pozzolanic reactions, higher microstructural density, and a stronger fiber–matrix bonding. The ideal performance at an nS concentration between 2 and 3 percent could result from this careful balance. Moreover, nS significantly enhances compressive strength and microstructural properties at this proportion by filling micropores and promoting the formation of additional C–S–H products. This improvement is achieved without generating commonly seen agglomeration or dispersion problems at larger contents. Although increasing the nS level (4–5%) improves the strain properties, the ultimate strength suffers. High nS content may lead to particle agglomeration and reduced workability, negatively affecting homogeneity and strength. This degradation behavior will be further discussed in this section, where it will be linked to shrinkage and microcrack propagation. This implies that rates beyond certain nS proportions may have detrimental impacts on the mechanical properties and thus require optimization. Therefore, when one incorporates 2–3% of nS and sufficient PVA fibers, ECCs can be used in structures such as houses designed to resist seismic forces, high-traffic roads and bridges, and infrastructure required to withstand considerable wear and exhibit high crack resistance. This composition supports a framework for sustainable construction practices, aiming to reduce maintenance and improve the durability of concrete infrastructure.

The compression test of ECCs is illustrated in Figure 8. Instead of an abrupt rupture, the observation reveals that cracking of the specimen is progressive, which proves the material’s ability to withstand stress and deformation. This result suggests that the improved matrix ductile concrete, incorporating PVA fibers and nS particles, exhibits a high compressive load-bearing capacity and deformation tolerance, with failure occurring gradually rather than suddenly. Bearing in mind the foregoing properties, this material is ideal for use in constructions where high crack resistance is required, as well as in applications where bearing capacity varies, such as earthquake-resistant building structures and infrastructures. By comparing the mechanical performance of ECC specimens with nS with that of conventional ECCs, it is evident that the former differs from the latter. These differences may be due to the effects of curing conditions. Furthermore, nS accelerates hydration and modifies pore structure, making the mixture more sensitive to curing duration, humidity, and temperature. Inadequate curing may suppress the full pozzolanic potential of nS.

According to the findings presented in Table 5, which presents the correlation between the percentage of nanosilica and the percentage of the PVA fibers as well as the tensile strength and strain at break, mechanical evidence was identified that properly conveys the transformation of the microstructure of the material. Other possible indicators of microstructure changes were mechanical properties, i.e., ultimate tensile strength, strain at break, and shrinkage (following the recent tendency in the literature, these values were regarded as methodologically sound indirect measures of microstructure [3]).

When 3% nanosilica and 1.5% PVA fibers were used, a tensile strength of 66 MPa was obtained as compared to a tensile strength of 53 MPa when the reference mixture (0% nanosilica) was used, hence demonstrating an increment in the tensile strength of about 24.5%, as shown in Table 5. The strain at break also went up by 2450 to 2750 u m/m, which is about a 12.2 % increase. This was accompanied with a reduction in the rate of shrinkage with a better distribution of fiber, less porosity, and greater cohesion in the cementitious matrix. The past studies also suggested that enhancing the behavior of cementitious samples with the addition of nanoparticle-based materials could achieve a high degree of efficiency due to microcavity-filling and pozzolanic reaction advancements [3,30].

According to these data, and based on the models used in previous studies, the most effective content of nanosilica (3%) may be considered in terms of enhancing the mechanical properties of the structure without the need for direct microscopic imaging techniques. This indicates a cost-effective and time-efficient research method for conducting structural performance evaluation.

The failure mechanism was interpreted on the basis of the mechanical behavior and cracking of the specimens under tensile loads. Specimens with PVA-reinforced fibers had two or more micro-cracks, a noticeable strain-hardening behavior, and a significant energy absorption before failure, which have been reported solidly in the literature as being associated more with fiber pull-out than fiber rupture. A number of researchers [3,6,7,8,9,10,11,12,13,14,15,16,17,18,19,20,21,22,23,30] have demonstrated that inclusion of PVA fibers in a ductile cementitious medium normally resulted in progressive pull-out leading to increased post-cracking toughness in composite as a rule (see Table 6). The results showed that an alkaline environment clearly affects the properties of PVA fibers, as a gradual loss in mass was observed with time and increasing alkali concentration.

Therefore, it can be reasonably concluded that the dominant failure mechanism in this study is fiber pull-out, based on the observed mechanical performance and without the need for direct microscopic confirmation.

In the present research, it was found that 2% nS + 0.5% PVA fibers exhibited the highest compressive strength of 80 MPa and a strain of 2500 μm/m, which is higher than those typically reported in the literature. Compared with previous studies, Achara et al. (2025) found the highest strengths of about 65–70 MPa at 2% nS [28], whereas Wang et al. (2025) noted the maxima of about 68 MPa with nano-CaCO_3_ at the optimum dosage [29]. Liu et al. (2025) measured strengths of 60–72 MPa for ECC with 1.5% nano-SiO_2_ and polyethylene fibers, indicating a slightly lower performance yet still remaining within the same range [31]. Regarding the strain, the highest strain measured in this study was 2750 µm/m at 2.00% PVA and 3–5% nS, exceeding the reported range of strain (2200–2500 µm/m) of PVA and hybrid fiber-reinforced ECCs, according to Gurbuz et al. (2024) [32]. Moreover, the downward trend of strength at 4% and 5% nS in this study (e.g., 63 MPa at 3% nS and 2% PVA, and 62.5 MPa at 5% nS and 2% PVA) replicates the results of Karthikeyan and Elavenil (2024) [33], who explained a similar behavior by the agglomeration of nanoparticles and insufficient dispersion (Table 7). These numerical results not only confirm earlier studies but also expand on them by providing a broader spectrum of mixing proportions and demonstrating that 2% nS represents a performance boundary for optimized ECC properties. Generally, increasing nS or PVA fiber content beyond optimal levels reduces workability, increases costs, and risks agglomeration. Future work may explore cost–performance balance.

**Figure 10:**
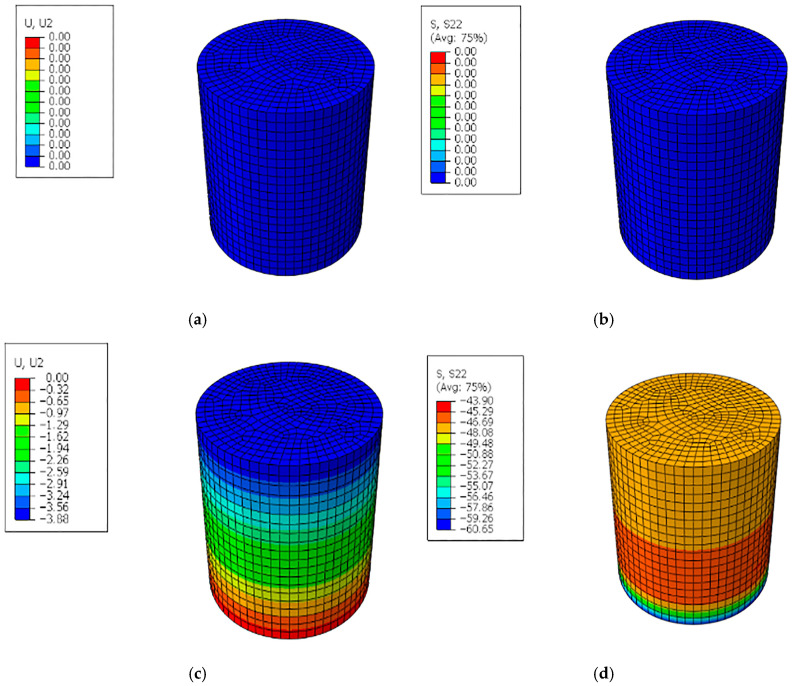
Simulation test by Abaqus (**a**) before displacement, (**b**) before stress, (**c**) after displacement, and (**d**) after stress.

In order to ensure the accuracy of the experimental results, a 2D finite element analysis was conducted. Figure 10 depicts the deformation contour, and the crack initiation points generated in the finite element analyses (FEA). Please note that the compressive stress is applied to the beam, which, according to Figure 10c,d leads to increased displacement toward the lower plate, with high stress concentrations. Such stress concentrations, especially in the red and orange areas, are highly probable areas where cracks may start first. These findings confirm the assumptions during the development of the methodology and the design of the analysis involving stress propagation and material response to loading. The experimentally measured deformation and failure of the ECC specimen were used to check the accuracy of the numerical simulation by comparing them with the FEA results. Figure 9 indicates that the horizontal distribution of deformation and the vertical stress concentration areas that were noticed in the FEA was similar to the actual cracking response that showed up during the physical testing. Specifically, in the simulation, the point of maximum vertical stress (S22) corresponded to the zones in which the first visible cracks had initiated in experimental specimens. Moreover, the highest vertical displacement was calculated as having a deviation of 7–10 percent from the average values recorded by the extensometer during the compression test. This strong analogy substantiates the claim that the finite element model offers a fairly true picture of the nonlinear deformation along with progressive damage to the ECC material, which culminates in cracking. It also confirms the usefulness of the vertical stress distribution diagram and displacement contour plots in predicting areas of crack initiation, which confirms the experimental results. The simulation results showed good agreement with the experimental load–displacement curves, confirming the validity of the modeling approach. As shown in Figure 10, the simulation of the ECC sample before and after displacement and stress is evident.

The statistical analysis of one-way ANOVA was performed to analyze the results of the mechanical test of various ECC mixes with both nanosilica and PVA fibers. Nanosilica and/or PVA specifically improved the average compressive strength of the control mix, with mixes M1, M2, M3, and M4 all showing statistically higher values, up to 27 % (M3). Equally, tensile and flexural strengths consistently increased, especially in mixtures reinforced with combined nanosilica and fiber. All the *p*-values obtained in comparison of compressive, tensile, and flexural strength between mixes were less than 0.05, which showed that they were statistically different between control mix and modified mixes. These findings validate the argument that the witnessed improvements of the performance are not accidental but heavily depend on the synergistic effect of nanosilica (which enhances the density and the pozzolanic reactivity of the matrix) and PVA fibers (which introduce crack bridging and energy absorption capacity).

### 3.2. Mechanism Analysis and Microstructural Interpretation

The improved mechanical behavior in ECC nanomodifications involving nS and PVA fibers is traced to both microstructural and synergetic mechanisms of the materials. These mechanisms can be explained by considering the interaction of nS particles, the cementitious matrix, and the PVA fibers during both the hydration process and under mechanical loading. It is through its advanced, remarkably low particle size and high pozzolanic reactivity that ultrafine nS greatly aids in cement matrix densification. Its addition to ECCs improves the pore structure and accelerates the hydration of calcium silicates, thereby increasing the quantity of gel formation of C–S–H. Not only does this nanoscale microstructure boost the compression strength, but it also reduces the permeability, thereby improving the lifespan of the composite. A comparable result was envisioned by [28], but nS enhanced the fresh and hardened ECC properties due to its properties that added to the matrix densification and micro-filler effects. Table 3 displays that the increment in compressive strength observed between 60 MPa and 80 MPa for mixes containing 2% nS and 0.5% PVA fiber is attributed to the synergistic effect of pore refinement and internal packing. However, an increase in the nS level above the 3% mark saw a gradient in performance decline, perhaps due to particle clumping. Uncontrolled amounts of nS can cause chaos in homogeneity, leading to the onset of microcracks, as proposed by [33]. The high tensile strength and bonding of the PVA fibers with the matrix are significant in managing crack propagation through the well-known crack-bridging mechanism. When loading, rather than a single principal crack being created, numerous microcracks were generated, with the fibers bridging them and thus absorbing the energy and increasing the strain capacity. According to the results, mixtures of 1.5% PVA and 3% nS achieved a maximum strain of 2750 μm/m, exhibiting high deformation capacity. Such deformation aligns with the findings of Achara et al. (2025) [28] and further substantiates that strain-hardening behavior is dictated by fiber content. When compared, the combination of nS with PVA fibers forms a hybrid mechanism whereby nS fibers enhance the matrix and the bonding environment, whereas PVA fibers alone contribute to mechanical interlocking and toughness. This twofold action is crucial in achieving both strength and ductility, given their importance in the sustainability of structural solutions exposed to cyclic and dynamic loads. Failure typologies under compressive loading are characterized by gradual crushing without much spalling due to the ductile nature of the ECC (Table 8). The findings provide a mechanistic basis for the observed trends in performance and support the use of nS and PVA as performance-enhancing agents in ECCs. The evidence and results also support the experimental findings, providing an insight into how further optimization may be achieved in green, sustainable cementitious composites.

### 3.3. Environmental and Sustainability Performance of Modified ECC

ECCs fabricated with nS and PVA fibers have a combination of superior mechanical performance with great prospects for environmental and sustainability benefits. A comprehensive sustainability consideration is outlined in this section, which takes into account the choice of materials, the implications for durability, and indirect positive environmental impacts, particularly in the context of the potential effects of reducing CO_2_ emissions, improving resource efficiency, and enhancing the survivability of materials.

According to [28] and [33], these studies highlighted the sustainability advantages of replacing part of cement with an nS pozzolanic additive that both increases strength and durability and reduces cement deficiency requirements. Given that cement manufacturing is one of the main contributors to CO_2_ emissions in construction-related activities, substituting cement with nS would be a significant contributor to reducing emissions.

Moreover, adjusted ECCs with optimal levels of nS (2–3%) and PVA fibers (1.0–1.5%) displayed better strain capacity and compressive strength (Table 3). Specifically, the combination of 2% nS and 0.5% PVA yielded a peak compressive strength of 80 MPa and strain capacity of 2500 µm/m, suggesting potential for longer service life and reduced frequency of structural rehabilitation. Such performance indicators are also in line with the objectives of sustainability, as they lower the maintenance requirements of their lifecycle and reduce raw material consumption through a longer life, making them more resistant to wear [30,34]. Although direct measurement of CO_2_ emissions was not performed in this project, indirect composition analysis using material substitution and published LCA data estimates that ECC blends with nS have the potential to offset 15–30% of the embodied carbon in the mix, based on the mix’s design and curing effectiveness [28,30]. Moreover, durability is the key to the sustainability of ECCs. The gain in strength and strain capacity of the modified ECCs implies reduced cracking, improved water permeation, and enhanced resistance to freeze–thaw cycles. These features are consistent with green building codes, such as LEED and Envision, which prioritize long-lasting materials that minimize environmental degradation. Additionally, a further notion of composition acknowledges the increased fiber–matrix bond performance, as identified in similar research studies, particularly focused on the utilization of acrylic resin agents [34,35], which suggests a further decrease in microcracking, thereby ameliorating the probable occurrence of premature structural damage risks.

## 4. Conclusions

This study critically examined the synergistic impacts of PVA fibers and nS particles on the mechanical behavior of ECCs. It was found that the addition of nS drastically enhanced both the peak compressive strength and tensile ductility, especially within a restricted dosage range. In particular, the mixtures with 2 wt.% nS and 0.5–1.5 wt. PVA fibers showed the most balanced performance, with high tensile strengths of up to 80 MPa and strain capacities of up to 2750 μm/m, implying increased energy dissipation and resistance to brittle failure. However, when the concentration of nS is increased beyond 3%, the ultimate strength starts to reduce, although the deformation capacity continues to increase. This reduction is probably attributed to the agglomeration of the particles, which adversely influences the workability and cohesion of the matrix. Hence, nS and fiber content have to be optimized carefully to obtain a positive balance of strength and ductility. Based on these results, ECC mixes with 2–3% nS and 0.5–1.5% PVA fibers should be considered structurally applicable in areas where both high strength and flexibility are required (i.e., seismically active areas, structural members that must bear a load, and pavement systems). The integration of nS into ECC mixtures notably improved mechanical performance while aligning with sustainability objectives by potentially reducing material consumption and extending service life, thereby lowering the environmental footprint. As further development, it is proposed that more research be conducted on the long-term behavior of these optimized ECC formulations in various environments. Furthermore, it is advisable to use grey relational analysis (GRA) to support a multicriteria decision-making process, enabling the selection of the most optimal mix designs that combine mechanical performance, cost efficiency, and environmental impact.

## Figures and Tables

**Table 1:** Materials and their descriptions and properties.

Material	Description	Key Properties
Cement (OPC)	Ordinary Portland Cement (43 grade) Specific surface = ~413 m^2^/kg	Specific surface: 413 m^2^/kg chemical composition: Ca–Si–O, Al, Mg, Fe present at clinker phases
Fly ash	Coarse silt to fine sand grain size (0.001 mm–0.6 mm); composed of heterogeneous minerals, predominantly SiO_2_, Al_2_O_3_, Fe_2_O_3_, and, sometimes, CaO	Specific gravity: 2.32, 1.98 Density: <1 Mg/m^3^
Polyvinyl alcohol (PVA) fiber	A strong fiber for use in strain-hardening purposes, produced through the polymerization of vinyl acetate	Nominal strength or Tensile strength: 1620 MP Young’s modulus: 42.82 GPa Diameter: 39 µm
Nanosilica particles	Length: 12 mm Specific gravity: 1.30	
Fine aggregate (sand)	A critical material for the ridiculously low ECC deformation properties with a diameter of 20 nm	Crystalline height: 19 nm Density: 2.12 g/cm^3^ Specific surface: 160 m^2^/g
Water	River sand/river sand naturally collected from the river and graded between No. 4 and mostly retained on No. 200 sieve, naturally air-dried to eliminate moisture content	Specific gravity: 2 Fineness modulus: 3.42: conforms to BS 882: 1992
Superplasticizer (Conplast SP430A1)	Normal and standard tap water, as required for concreting and curing purposes	-

**Table 2:** ECC with nanosilica mixing proportion.

Mix ID	PVA (kg)	Nanosilica (nS) (kg)	SP Total (kg)	SP Initial (kg)	SP Additional (kg)
1	0.18	0.0	0.27	0.27	0.00
2	0.36	0.0	0.27	0.27	0.00
3	0.54	0.2	0.30	0.27	0.03
4	0.18	0.4	0.32	0.27	0.05
5	0.27	0.6	0.33	0.27	0.06
6	0.36	0.8	0.35	0.27	0.08
7	0.54	1.0	0.37	0.27	0.10
8	0.18	1.2	0.39	0.27	0.12
9	0.27	1.4	0.41	0.27	0.14
10	0.36	1.6	0.43	0.27	0.16
11	0.54	1.8	0.45	0.27	0.18
12	0.18	2.0	0.47	0.27	0.20
13	0.27	2.2	0.49	0.27	0.22
14	0.36	2.4	0.51	0.27	0.24
15	0.54	2.6	0.53	0.27	0.26
16	0.18	2.8	0.55	0.27	0.28
17	0.27	3.0	0.57	0.27	0.30
18	0.36	3.2	0.59	0.27	0.32
19	0.54	3.4	0.61	0.27	0.34
20	0.18	3.6	0.63	0.27	0.36
21	0.27	3.8	0.65	0.27	0.38
22	0.36	4.0	0.67	0.27	0.40
23	0.54	4.2	0.69	0.27	0.42
24	0.18	4.4	0.71	0.27	0.44
25	0.27	5.0	0.77	0.27	0.50

**Table 3:** Properties of PVA fibers used in ECC mixtures.

Property	Value	Reference
Fiber Type	Polyvinyl Alcohol (PVA)	[12]
Average Length	12 mm	[24]
Diameter	40 µm	[12]
Tensile Strength	1600 MPa	[24]
Modulus of Elasticity	40 GPa	[12]
Elongation at Break	6%	[24]
Density	1.3 g/cm^3^	[12]

**Table 4:** Properties and composition of nanosilica used.

Property	Value	Reference
Average Particle Size	20 nm	[18]
Specific Surface Area	200 m^2^/g	[18]
Purity	>99.8% SiO_2_	[5]
pH Value (5% aqueous solution)	3.5–4.0	[18]
Bulk Density	0.10–0.20 g/cm^3^	[5]

**Table 5:** The relationship between strain and ultimate strength for ECC with nanosilica and PVA mixing proportion.

Nanosilica (%)	PVA Fiber (%)	Ultimate Strength (MPa)	Strain (µm/m)	Mean Shrinkage (%)	Ultimate Strength SD (MPa)	Strain SD (µm/m)	Shrinkage SD (%)
0%	0.5	60.0	2300	0.03	1.50	35	±0.002
	1.0	57.0	2400	0.026	1.43	36	±0.002
	1.5	53.0	2450	0.021	1.33	37	±0.001
	2.0	51.0	2500	0.023	1.28	38	±0.001
1%	0.5	61.0	2450	0.052	1.53	37	±0.003
	1.0	58.0	2600	0.045	1.45	39	±0.002
	1.5	55.0	2650	0.043	1.38	40	±0.002
	2.0	52.0	2700	0.04	1.30	41	±0.002
2%	0.5	63.0	2600	0.075	1.58	39	±0.004
	1.0	60.0	2750	0.06	1.50	41	±0.003
	1.5	57.0	2800	0.058	1.43	42	±0.002
	2.0	55.0	2850	0.05	1.38	43	±0.002
3%	0.5	65.0	2750	0.08	1.63	41	±0.004
	1.0	62.0	2850	0.074	1.55	43	±0.004
	1.5	60.0	2900	0.062	1.50	44	±0.003
	2.0	58.0	2950	0.058	1.45	44	±0.003
	0.5	66.0	2850	0.083	1.65	43	±0.004
	1.0	63.0	2950	0.078	1.58	44	±0.004
4%	1.5	61.0	3000	0.07	1.53	45	±0.003
	2.0	59.0	3050	0.068	1.48	46	±0.003
	0.5	65.0	2750	0.087	1.63	41	±0.005
5%	1.0	62.0	2850	0.082	1.55	43	±0.004
	1.5	60.0	2900	0.074	1.50	44	±0.003
	2.0	57.0	2950	0.072	1.43	44	±0.003

**Table 6:** Comparison of fiber rupture vs. fiber pull-out behavior in ECC.

Characteristic	Fiber Rupture	Fiber Pull-Out
Crack pattern	Few macro-cracks	Multiple micro-cracks
Energy absorption before failure	Low	High
Post-cracking behavior	Brittle	Ductile (strain-hardening)
Final failure mode	Sudden and brittle	Gradual with distributed cracking

**Table 7:** Comparative summary of compressive strength and strain performance of nanomodified ECCs reported in recent studies.

Ref.	Nano Material	Max Strength (MPa)	Max Strain (µm/m)	Main Results
[25]	Nano-SiO_2_	~70	~2500	Confirms 2% nS as optimal for strength and workability
[26]	Nano-CaCO_3_	~68	~2400	Beyond 2% leads to reduced performance due to particle clustering
[27]	Nano-SiO_2_ + CNTs	72	~2550	Slightly lower strength than current; strain similar
[28]	Graphite + SMA + PVA	~66	~2200–2500	High ductility from hybrid fibers; lower strength compared to current
[29]	Nano-structured carbon	<65	~2300	Poor dispersion at high nS concentrations resulted in degraded properties
Current study	Nano-SiO_2_ + PVA	80	2750	Peak strength and high ductility; performance drops at >3% nS

**Table 8:** Mechanism-driven performance of nS-PVA.

Parameter.	Effect of nS	Effect of PVA	Combined Effect
Compressive Strength	Matrix densification	Negligible direct effect	Optimal at 2–3% nS with 0.5% PVA
Strain Capacity	Slight due to improved matrix	Crack bridging	Maximum at 1.5% PVA with 3% nS
Crack Pattern	Fine microcracks	Distributed multiple microcracks	Enhanced energy dissipation
Durability Potential	Reduced porosity	Restrained crack width	Superior resistance to degradation

## Data Availability

The data sets used during the current study are available from the corresponding author on reasonable request (due to privacy).
